# MYCBP2 expression correlated with inflammatory cell infiltration and prognosis immunotherapy in thyroid cancer patients

**DOI:** 10.3389/fimmu.2022.1048503

**Published:** 2022-12-13

**Authors:** Guilin Wang, Chen Miao, Lijun Mo, Ulf D. Kahlert, Jinfeng Wu, Minglin Ou, Renxiang Huang, Ruifa Feng, Weiyi Pang, Wenjie Shi

**Affiliations:** ^1^Breast Center, The Second Affiliated Hospital of Guilin Medical University, Guilin, Guangxi, China; ^2^Department of Pathology, The First Affiliated Hospital of Nanjing Medical University, Nanjing, China; ^3^Molecular and Experimental Surgery,University Clinic for General, Visceral, Vascular and Transplantation Surgery, Faculty of Medicine, Otto-von-Guericke-University, Magdeburg, Germany; ^4^Guangxi Key Laboratory of Environmental Exposomics and Entire Lifecycle Heath, Guilin Medical University, Guilin, Guangxi, China; ^5^University Hospital for Gynecology, Pius-Hospital, University Medicine Oldenburg, Oldenburg, Germany

**Keywords:** immune checkpoint inhibitors, MYCBP2, myc binding protein 2, prognosis, thyroid cancer

## Abstract

**Introduction:**

Immune checkpoint inhibitors (ICIs) have shown promising results for the treatment of multiple cancers. ICIs and related therapies may also be useful for the treatment of thyroid cancer (TC). In TC, Myc binding protein 2 (MYCBP2) is correlated with inflammatory cell infiltration and cancer prognosis. However, the relationship between MYCBP2 expression and ICI efficacy in TC patients is unclear.

**Methods:**

We downloaded data from two TC cohorts, including transcriptomic data and clinical prognosis data. The Tumor Immune Dysfunction and Exclusion (TIDE) algorithm was used to predict the efficacy of ICIs in TC patients. MCPcounter, xCell, and quanTIseq were used to calculate immune cell infiltration scores. Gene set enrichment analysis (GSEA) and single sample GSEA (ssGSEA) were used to evaluate signaling pathway scores. Immunohistochemical (IHC) analysis and clinical follow up was used to identify the MYCBP2 protein expression status in patients and associated with clinical outcome.

**Results:**

A higher proportion of MYCBP2-high TC patients were predicted ICI responders than MYCBP2-low patients. MYCBP2-high patients also had significantly increased infiltration of CD8+ T cells, cytotoxic lymphocytes (CTLs), B cells, natural killer (NK) cells and dendritic cells (DC)s. Compared with MYCBP2-low patients, MYCBP2-high patients had higher expression of genes associated with B cells, CD8+ T cells, macrophages, plasmacytoid dendritic cells (pDCs), antigen processing and presentation, inflammatory stimulation, and interferon (IFN) responses. GSEA and ssGSEA also showed that MYCBP2-high patients had significantly increased activity of inflammatory factors and signaling pathways associated with immune responses.In addiation, Patients in our local cohort with high MYCBP2 expression always had a better prognosis and greater sensitivity to therapy while compared to patients with low MYCBP2 expression after six months clinic follow up.

**Conclusions:**

In this study, we found that MYCBP2 may be a predictive biomarker for ICI efficacy in TC patients. High MYCBP2 expression was associated with significantly enriched immune cell infiltration. MYCBP2 may also be involved in the regulation of signaling pathways associated with anti-tumor immune responses or the production of inflammatory factors.

## Introduction

Thyroid cancer (TC) originates from follicular epithelial cells or parafollicular epithelial cells of the thyroid. It is the most common malignant tumor in endocrine system and comprises about 90% of endocrine cancers (1, 2). Although most TCs are relatively inert and can be effectively treated by surgery, radiation,131 and thyroid stimulating hormone (TSH) inhibition therapy, some cases are refractory and eventually lead to disease progression, recurrence, and even death (3). Although targeted therapies such as kinase inhibitors can prolong patient prognosis, the effectiveness of kinase inhibitors is severely limited due to the rapid development of drug resistance and the occurrence of adverse reactions (4–8). In recent years, with increasing use of immune checkpoint inhibitors (ICIs) for anti-cancer therapy, research on the use of ICIs for TC is also rapidly advancing, with the hope of improving the prognosis of TC patients.

Previous research has shown that biomarkers predicting the efficacy of ICIs can help to screen patients and further improve prognoses (9, 10). At present, Programmed Cell Death-Ligand 1 (PD-L1) expression and tumor mutation burden (TMB) are the main markers of ICI effectiveness (9–12). However, these biomarkers also have some disadvantages: thresholds of TMB are different across studies, and PD-L1 detection platforms vary (13–16). Therefore, there is need to identify biomarkers that more comprehensively predict the efficacy of ICIs, allowing for the development of tumor precision medicine.

Myc binding protein 2 (MYCBP2) is a E3 ubiquitin ligase (17). Previous studies have shown that MYCBP is associated with the occurrence and prognosis of lung cancer, gastric cancer, breast cancer, and glioma (18–20). Pierre et al. found that MYCBP2 expression was associated with an M2-like macrophage phenotype in a mouse model (21). Schaid et al. found that MYCBP2 expression was associated with an elevated risk of invasive prostate cancer (22). However, the relationship between MYCBP2 expression and ICI efficacy in TC remains unclear.

In the present study, we downloaded data on a TC cohort from the Gene Expression Omnibus (GEO) database and a thyroid carcinoma (THCA) cohort from the Cancer Genome Atlas (TCGA) database. We used the Tumor Immune Dysfunction and Exclusion (TIDE) algorithm to predict the therapeutic effect of ICIs in TC patients. Next, we evaluated the relationship between MYCBP2 expression and ICI efficacy in TC patients and explored its potential molecular mechanism.

## Methods

### THCA cohort and ICIs datasets

We downloaded a THCA cohort (GSE138042-THCA) with transcriptomic data from the GEO database (https://www.ncbi.nlm.nih.gov/geo/) (23). We also downloaded another THCA cohort (TCGA-THCA) with gene expression profile data from TCGA database (https://portal.gdc.cancer.gov/) (24). Because the public databases did not publish data on the efficacy of ICIs by THCA, we used the TIDE algorithm (http://tide.dfci.harvard.edu) (25). The transcriptomic data from GSE138042-THCA and TCGA-THCA were used to predict the response of each patient to ICIs, and each patient was categorized as a responder or non-responder. We also downloaded data from an ICI-treated melanoma cohort (26) and an ICI-treated urothelial cancer (UC) (27) cohort, including transcriptomic data and prognostic data from the cbioportal webtools (https://www.cbioportal.org/) (28). Also, we downloaded two datasets related to the mice model treated with the ICIs [GSE146027 (29) and GSE151829 (30)]. We downloaded the expression data and survival data of different cancer types [GSE26304 (31), GSE1456 (32), GSE5327 (33), GSE30929 (34), GSE26939 (35), GSE30219 (36), GSE41271 (37), GSE30219 (36), GSE37745 (38), GSE50081 (39), GSE62452 (40), and GSE16560 (41)] to further validate the relationship between the expression of MYCBP2 and the survival time.

### Analysis of tumor immune microenvironment

We downloaded the gene set of immune checkpoint (IC) related molecules and immune related molecules from CAMOIP (http://camoip.net/) (42). We used three immune cell evaluation algorithms (1.MCPcounter, 2.xCell, and 3.quanTIseq) to calculate the immune cell infiltration score of each THCA patient from the expression data (43–45).

### Pathway enrichment analysis

We used gene set enrichment analysis (GSEA) to analyze the transcriptomic data of THCA patients and obtained pathway enrichment scores and P values of pathways from the Kyoto Encyclopedia of Genes and Genomes (KEGG) (https://www.kegg.jp/), Reactome (https://reactome.org/), Gene Ontology- Biological Process (GO-BP), Gene Ontology- Cellular Component (GO-CC), and Gene Ontology- Molecular Function (GO-MF) databases (46). We also applied the single sample GSEA (ssGSEA) algorithm to analyze the transcriptomic data of each THCA patient and then obtained the enrichment scores of each patient in the Molecular Signatures Database (MsigDB) database (http://www.gsea-msigdb.org/gsea/downloads.jsp) (47).

### Immunohistochemical analysis and clinical follow up

To identify the MYCBP2 protein expression status in patients and associated with clinical outcome. We collect tissues samples from thyroid cancer patients,who accepted surgery operation in Breast Center of The Second Affiliated Hospital of Guilin Medical University, to conduct immunohistochemical analysis and follow up six months to evaluate the prognosis difference between high and low expression groups. Thirty-six tissues from twelve patients, each patient has three tissues obtained from different surgery sections, and these tissues were washed with Phosphate-Buffered Saline (PBS) and then incubated with 3% H2O2 for 10 minutes. Samples were incubated with antibody against MYCBP2 (1 mg/mL diluted 1:200, 27951, Proteintech, CA) at room temperature for two hours. After incubation with polymer enhancer for 20 minutes, the tissue was incubated with polymer enhancer and enzyme-labeled rabbit polymers. Slides were washed with PBS and fresh diaminobenzidine and counterstained with hematoxylin; antigen retrieval was performed using 0.1% HCl, and slides were then dehydrated with ethanol, cleaned with xylene, and fixed with neutral balata. The images were observed and photographed using a fluorescence microscope and visualized under a light microscope at 100× magnification by a blinded observer. Light to dark brown staining indicated a positive result from low to high. The stained areas were analyzed using Image J. Patients who donated samples for IHC also accepted clinical follow up to evaluate the association between MYCBP2 expression and patient outcomes following surgery. All patient and tissue studies were approved by the Ethics Committee of Breast Center of The Second Affiliated Hospital of Guilin Medical University.

### Statistical analysis

The Mann-Whitney U test was used to compare differences between the MYCBP2-high and MYCBP2-low groups in continuous variables. Fisher’s exact test was used to compare differences between the two groups in categorical variables. Kaplan-Meier curves were used to visualize differences in overall survival (OS) time between the two groups, and a log rank test was used to calculate the difference in OS time. We used the ggplot2 R package to generate boxplots. Visualization and data analysis for the present study were conducted in Rstudio software (Version 4.1.2). P values were bilateral and P values < 0. 05 were considered statistically significant.

## Results

### Relationship between MYCBP2 expression and ICI efficacy in THCA

**Figure 1:**
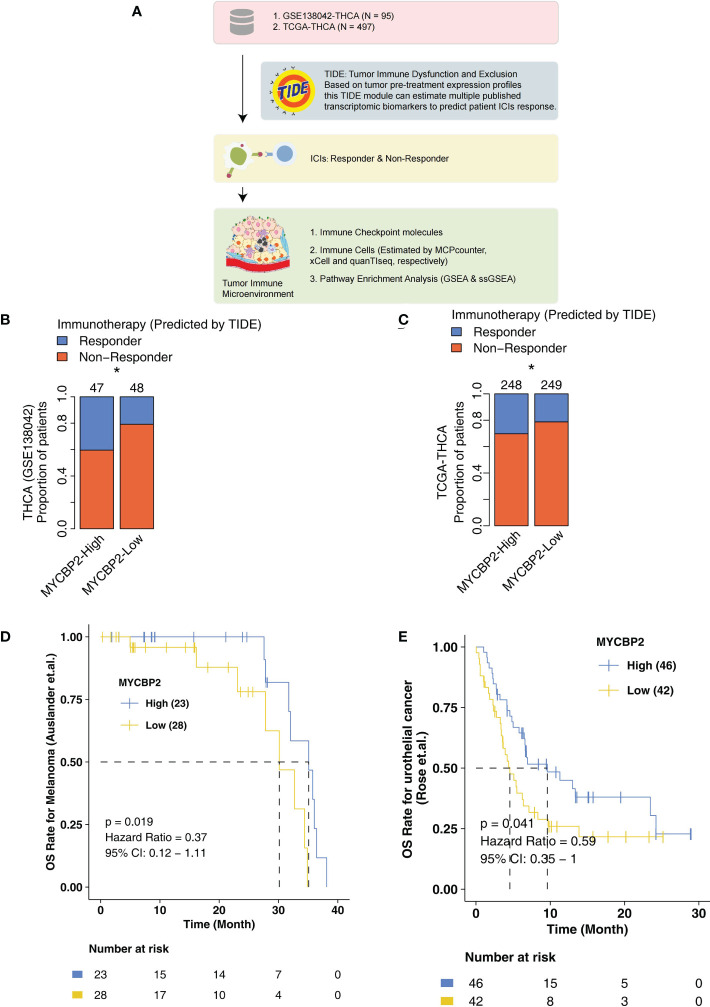
The association between the MYCBP2 expression and ICI efficacy. **(A)** The overall design of the study. The differences in predicted ICI efficacy between the MYCBP2-High and MYCBP2-Low TC patients in the GSE138042-TC **(B)** and TCGA-THCA **(C)** cohorts. KM curve showing the differences in the prognoses of ICI-treated patients between the MYCBP2-High and MYCBP2-Low groups in the melanoma **(D)** and urothelial cancer **(E)** cohorts. (*: P < 0.05).

In the present study, we explored the relationship between MYCBP2 expression and ICI efficacy in THCA patients and analyzed potential molecular mechanisms for this relationship (**Figure 1A**). In the GSE138042-THCA cohort, MYCBP2-high patients receiving ICIs had a higher proportion of predicted responders than MYCBP2-low patients receiving ICIs (evaluated by the TIDE algorithm) (**Figure 1B**, P < 0. 05). MYCBP2-high patients in TCGA-THCA cohort also had a higher predicted response rate to ICIs (**Figure 1C**, P < 0. 05). Next, we used the ICI-treated melanoma and urothelial cohorts to further verify the relationship between MYCBP2 expression and prognosis in ICI-treated patients (**Figures 1D, E**). **Figure 1D** shows that MYCBP2-high melanoma patients had significantly longer OS time than MYCBP2-low patients (log rank P = 0. 019, HR = 0. 3). **Figure 1E** shows that MYCBP2-high urothelial cancer patients had a significantly longer OS time than MYCBP2-low patients (log rank P = 0. 041; HR = 0. 59, 95% CI: 0. 35-1). In the mice model, we found that the expression of Mycbp2 was significantly increased in the ICIs-responders compared with ICIs-non-responders (**Supplementary Figures 1A, B**). Also, in the multiple cancer types, we found that MYCBP2-high patients had significantly prolonged survival time compared with MYCBP2-low patients (**Supplementary Table 1**, **Supplementary Figure 2**).

### Relationship between MYCBP2 expression, IC-related molecules, and immune related genes

**Figure 2:**
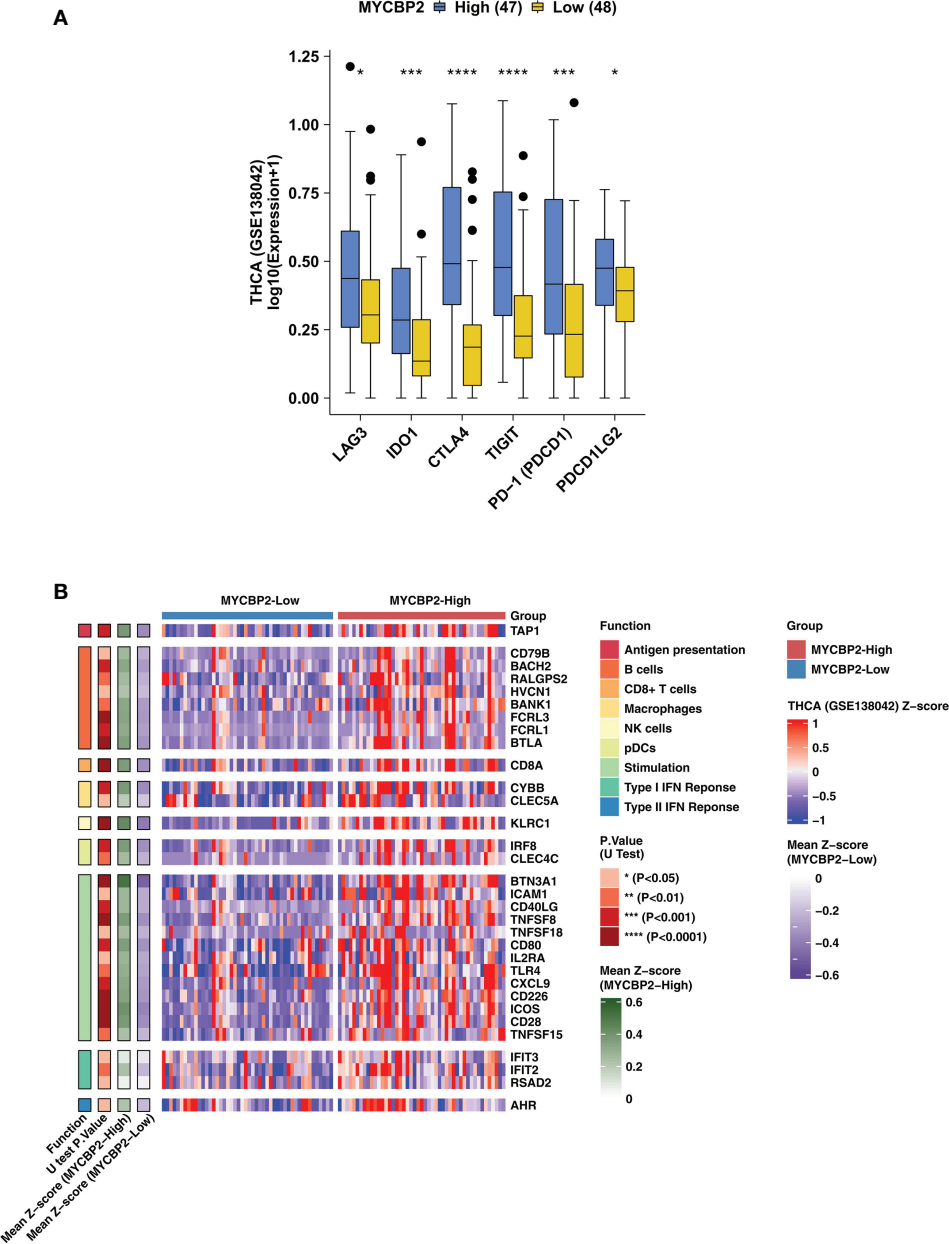
The association between the MYCBP2 expression and immune-related gene expression. **(A)** Differences in the expression of immune checkpoint molecules between MYCBP2-High and MYCBP2-Low patients in the GSE138042-TC cohort. **(B)** Heatmap depicting differences the expression of immune-related genes (antigen presentation, B cells, CD8+ T cells, macrophages, NK cells, pDCs, stimulation, and IFN responses) between MYCBP2-High and MYCBP2-Low patients in the GSE138042-TC cohort. (*: P < 0.05; ***: P < 0.001; ****: P < 0.0001).

In TCGA-THCA cohort, MYCBP2-high patients had significantly higher expression of IC-related molecules, such as LAG3, IDO1, CTLA4, TIGIT, PD-1 and PDCD1LG2, than MYCBP2-low patients (**Figure 2A**, P < 0. 05). In the GSE138042-THCA cohort, the heatmap also revealed differences between MYCBP2-high and MYCBP2-low patients in the expression of genes associated with antigen presentation, B cells, CD8+ T cells, macrophages, pDCs, inflammatory stimulation, and IFN responses (**Figure 2B**). In the TCGA-THCA cohort, these genes were expressed at significantly higher levels in MYCBP2-high patients than in MYCBP2-low patients (**Supplementary Figure 3**).

### Relationship between MYCBP2 expression and immune cell infiltration

**Figure 3:**
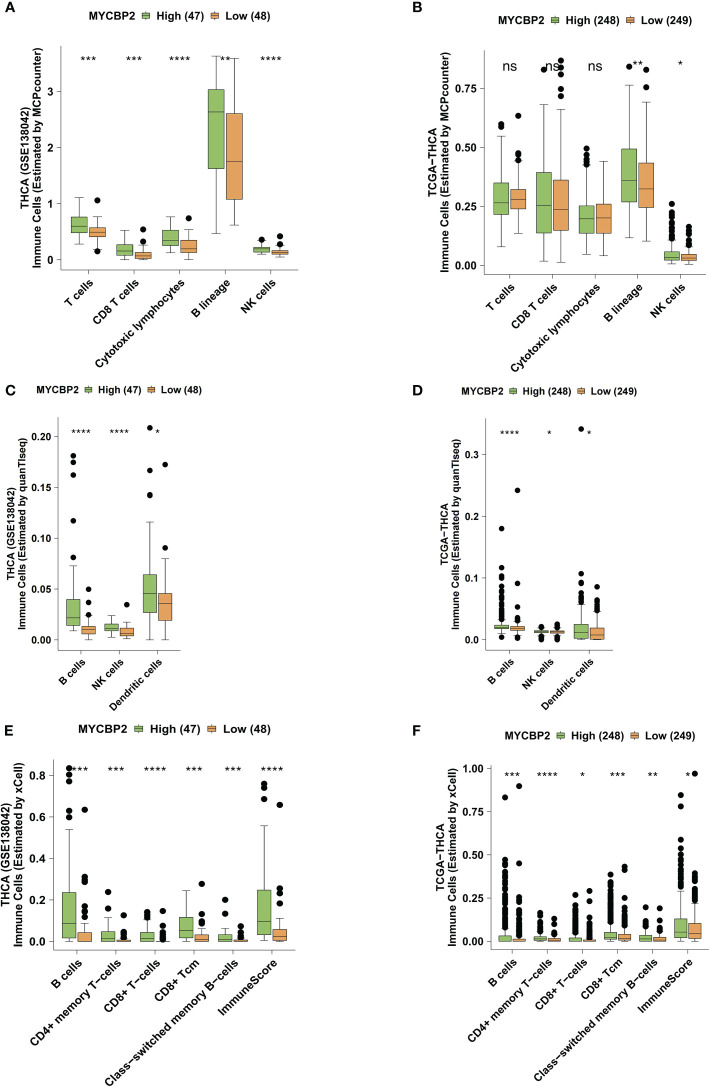
The association between the MYCBP2 expression and immune cell infiltration. Differences in tumor infiltrating immune cells estimated by MCPcounter, between the MYCBP2-High and MYCBP2-Low TC patients in the GSE138042-TC **(A)** and TCGA-THCA **(B)** cohorts. Differences in tumor infiltrating immune cells estimated by quanTIseq, between the MYCBP2-High and MYCBP2-Low TC patients in the GSE138042-TC **(C)** and TCGA-THCA **(D)** cohorts. Differences in tumor infiltrating immune cells estimated by xCell, between the MYCBP2-High and MYCBP2-Low TC patients in the GSE138042-TC **(E)** and TCGA-THCA **(F)** cohorts. (ns: not significant; *: P < 0.05; **: P < 0.01; ***: P < 0.001; ****: P < 0.0001).

We used the MCPcounter algorithm to evaluate immune cell infiltration in the TIME of THCA patients (**Figures 3A, B**). In both the GSE138042-THCA (**Figure 3A**) and TCGA-THCA (**Figure 3B**) cohorts, MYCBP2-high patients had significantly higher infiltration of CD8+ T cells, cytotoxic lymphocytes (CTLs), B cells, and NK cells than MYCBP2-low patients. The quanTIseq algorithm was also used to verify immune cell infiltration fractions (**Figures 3C, D**). Using the quanTIseq algorithm, we found that MYCBP2-high patients had significantly enriched B cells, NK cells, and DCs (**Figure 3C**: GSE138042-THCA; **Figure 3D**: TCGA-THCA, all P < 0.05). We also used the xCell algorithm to evaluate the proportions of tumor infiltrating immune cells (**Figures 3E, F**). Compared with MYCBP2-low patients, MYCBP2-high patients had significantly increased B cells, memory CD4+ T cells, CD8+ T cells, central memory CD8 + T cells (TCMs), and class-switched memory B-cells (**Figure 3E**: GSE138042-THCA; **Figure 3F**: TCGA-THCA).

### Relationship between MYCBP2 expression and immune signaling pathways

**Figure 4:**
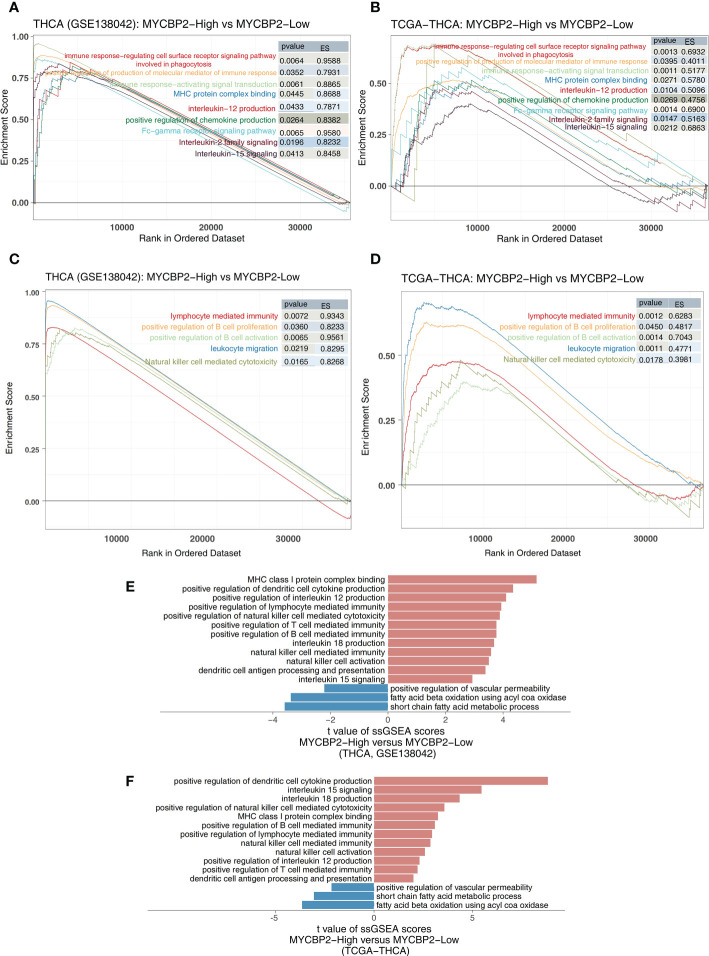
The association between the MYCBP2 and immune-related signaling pathways. Differences in inflammatory molecule related signaling pathways between MYCBP2-High and MYCBP2-Low TC patients in the GSE138042-TC **(A)** and TCGA-THCA **(B)** cohorts. Differences in immune response related signaling pathways between the MYCBP2-High and MYCBP2-Low TC patients in the GSE138042-TC **(C)** and TCGA-THCA **(D)** cohorts. Barplot showing the differences in ssGSEA scores between the MYCBP2-High and MYCBP2-Low TC patients in the GSE138042-TC **(E)** and TCGA-THCA **(F)** cohorts.

We found that MYCBP2-high patients had higher activation of immune signaling pathways than MYCBP2-low patients (**Figure 4A**: GSE138042-THCA; **Figure 4B**: TCGA-THCA, ES > 0, P < 0. 05), including pathways regulating cell surface receptor signaling and phagocytosis, Fc receptor signaling, signal transduction, chemokine production, molecular mediators of immune responses, interleukin-12 (IL-12) production, MHC protein complex binding, interleukin-15 (IL-15) signaling, and interleukin-2 (IL-2) family signaling. Signaling pathways related to immune responses and immune cell activation were also significantly upregulated in MYCBP2-high patients, including B cell activation, lymphocyte mediated immunity, leukocyte migration, B cell proliferation, and NK cell mediated cytotoxicity (**Figure 4C**: GSE138042-THCA; **Figure 4D**: TCGA-THCA, ES > 0, P < 0. 05). The results of ssGSEA showed that MYCBP2-high patients had higher immune response pathway scores than MYCBP2-low patients, including pathways for MHC class I protein complex binding, DC cytokine production, MHC class IB receptor activity, IL-12 production, lymphocyte mediated immunity, NK cell mediated cytotoxicity, T cell mediated immunity, interleukin-18 (IL-18) production, NK cell mediated immunity, NK cell activation, DC antigen processing and presentation, and IL-15 signaling (**Figure 4E**: GSE138042-THCA; **Figure 4F**: TCGA-THCA, P < 0. 05). On the contrary, lipid metabolism related pathways (such as short chain fatty acid metabolism, fatty acid beta oxidation using acyl COA oxidase, and positive regulation of vascular permeability) were significantly downregulated in MYCBP2-high patients compared to MYCBP2-low patients (**Figure 4E**: GSE138042-THCA; **Figure 4F**: TCGA-THCA, P < 0. 05).

### Validation of the local cohort based on MYCBP2 status

**Figure 5:**
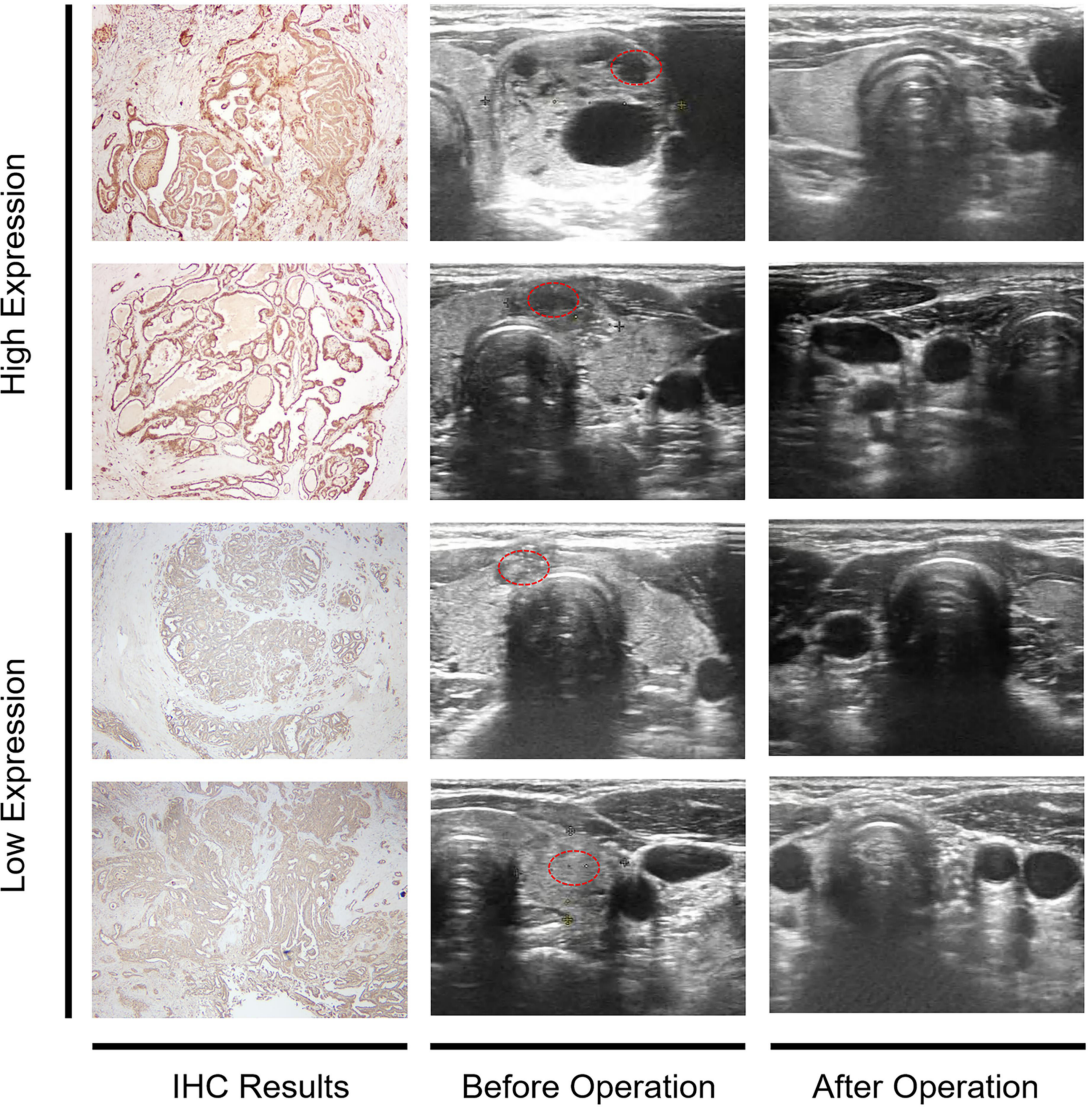
IHC analysis of MYCBP2 expression and clinical follow up of TC patients based on their MYCBP2 expression status.Here, we show four representive IHC results of patients and corresponding ultrasound follow-up image.

IHC revealed MYCBP2 expression levels in TC tissues. Patients in our local cohort with high MYCBP2 expression always had a better prognosis and greater sensitivity to therapy compared to patients with low MYCBP2 expression after six months clinic follow up (**Figure 5**).

## Discussion

Compared to conventional chemotherapy, ICIs has higher safety and fewer adverse reactions, making it a useful treatment regimen for various advanced cancers (48). ICIs is now a potential treatment for patients with advanced and refractory TC (49–51). In this study, we found that higher MYCBP2 expression was associated with better outcomes in TC patients receiving ICIs. Immune cell infiltration analysis showed that higher MYCBP2 expression was associated with significantly enriched inflammatory immune cell infiltration and higher expression of inflammatory molecules. Pathway enrichment analysis showed that MYCBP2 may be involved in anti-tumor immune responses. Therefore, MYCBP2 may be a novel biomarker for ICI efficacy in TC patients.

The significantly enriched inflammatory immune cell infiltration in MYCBP2-high patients is a potential molecular mechanism for the greater efficacy of ICIs in these patients. Bastman et al. found that some TC patients with greater programmed death 1 [PD-1(+)] CD4+ T cell and PD-1(+) CD8+ T cell infiltration could benefit from anti-PD-1/PD-L1 treatment (52). CD8 + T cells can kill target cells in an antigen-specific manner, and improve the disease-free survival (DFS) rate of patients (53). NK cells also play a key role in anti-tumor immune monitoring. NK cells can not only kill tumor or infected cells directly, but they can also indirectly enhance antibody and T-cell-mediated anti-tumor immune responses (54). NK cells also release lysosomes containing perforin and granzyme to kill target cells (55). Many studies have also shown that DCs are a key regulatory factor for the efficacy of ICIs and other tumor immunotherapies (56). Accordingly, researchers are now attempting to engineer DCs to activate and drive T cells into the TIME, particularly for tumors with weak immunogenicity (non-inflammatory of “cold” tumors), to improve the efficacy of ICIs (51, 57). In our study, we found that MYCBP2-high patients had significantly increased CD8+ T cells, B cells, NK cells, and DCs in the TIME compared to MYCBP2-low patients.

The high expression of inflammatory molecules and activation of immune response pathways in MYCBP2-high patients is another potential molecular mechanism for the greater ICI efficacy in MYCBP2-high patients. IL-15 can maintain NK cell populations and tumor killing ability in the TIME (58). Recently, a clinical trial showed that IL-15 can increase CD8+ T cell and NK cell numbers up to 5.8 and 38 times that of the control group, respectively, and 17 of the 27 patients treated with IL-15 showed no tumor progression (59). Several other studies have attempted to activate NK cells for immunotherapy using cytokines. For example, IL-21 has been shown to improve the exhaustion status of the NK cells (60), and the combination of IL-12, IL-15, and IL-18 can increase memory NK cells and stimulate IFN-γ secretion (61, 62). In our study, we found that MYCBP2-high patients had significantly upregulated IL-12 production, IL-15 signaling, IL-2 family signaling, and IL-18 signaling compared to MYCBP2-low patients.

The pattern of fatty acid metabolism in TME is also crucial in the immunomodulatory function of tumor tissues and immunotherapy tolerance. It has been shown that the expression of genes for fatty acid oxidation is upregulated in Treg and that the level of fatty acid oxidation is increased, thus promoting Treg production (63). The rate of *de novo* synthesis of the fatty acid in tumor cells is usually increased, thus shifting energy production to anabolic pathways for the production of plasma membrane phospholipids and signaling molecules (64). Lipid accumulation in tumor-infiltrating myeloid cells has been shown to predispose these immune cells to an immunosuppressive and anti-inflammatory phenotype through metabolic reprogramming (65, 66). In our study, we found that lipid metabolism related pathways (such as short chain fatty acid metabolism, fatty acid beta oxidation using acyl COA oxidase, and positive regulation of vascular permeability) were significantly downregulated in MYCBP2-high patients compared to MYCBP2-low patients.

Some limitations are not ignored in our study, first, clinical long-time follow-up was not conducted, and in the future, a more clinical trial should be considered to demonstrate this gene’s clinical value, and in addition, Due to experimental constraints, organoid-related experiments have not been considered, which is a regret, and we hope we will complete this procedure in future cooperation with another excellent laboratory.

## Conclusions

In summary, we found that MYCBP2-high TC patients had better responses to ICIs than MYCBP2-low patients, with a higher proportion of predicted ICI responders in the MYCBP2-high group. Higher MYCBP2 expression was associated with significantly enriched inflammatory immune cell infiltration and higher immune response pathway activity.

## Data availability statement

The datasets presented in this study can be found in online repositories. The names of the repository/repositories and accession number(s) can be found in the article/**Supplementary Material**.

## Ethics statement

The studies involving human participants were reviewed and approved by The Ethics Committee of Breast Center of The Second Affiliated Hospital of Guilin Medical University. The patients/participants provided their written informed consent to participate in this study.

## Author contributions

Conceptualization, RF, WP, WS. Formal analysis, GW, CM, LM, UK. Software, GW, CM, LM, UK. Supervision, GW, CM, LM. Resources, GW, CM, LM, UK. Visualization, GW, CM, LM, UK. Writing–original draft, JW, MO, RH. Writing–review & editing, GW, CM, LM, JW, MO, RH, RF, WP, WS. All authors contributed to the article and approved the submitted version.

## Supplementary material

Supplementary Figure 1 The differences of the expression of the Mycbp2 between the ICIs-responders and ICIs-non-responders in the GSE146027 **(A)** and GSE151829 **(B)**.

Supplementary Figure 2 The Kaplan-Meier curves depicting differences in the prognoses of cancer patients between the MYCBP2-High and MYCBP2-Low groups (GSE26304, GSE1456, GSE5327, GSE30929, GSE26939, GSE30219, GSE41271, GSE30219, GSE37745, GSE50081, GSE62452, and GSE16560).

Supplementary Figure 3 Heatmap depicting differences in the expression of immune-related genes (antigen presentation, B cells, CD8+ T cells, macrophages, NK cells, pDCs, stimulation, and IFN responses) between MYCBP2-High and MYCBP2-Low patients in TCGA-THCA.

Supplementary Table 1 The Kaplan-Meier result of the relationship between the expression of MYCBP2 and survival times of the different cancer patients (GSE26304, GSE1456, GSE5327, GSE30929, GSE26939, GSE30219, GSE41271, GSE30219, GSE37745, GSE50081, GSE62452, and GSE16560).
