# Supplementary figures and images for: MYCBP2 expression correlated with inflammatory cell infiltration and prognosis immunotherapy in thyroid cancer patients
Source: Front Immunol. 2022 Dec 13;13:1048503. doi: 10.3389/fimmu.2022.1048503 (PMC9792662; doi:10.3389/fimmu.2022.1048503)

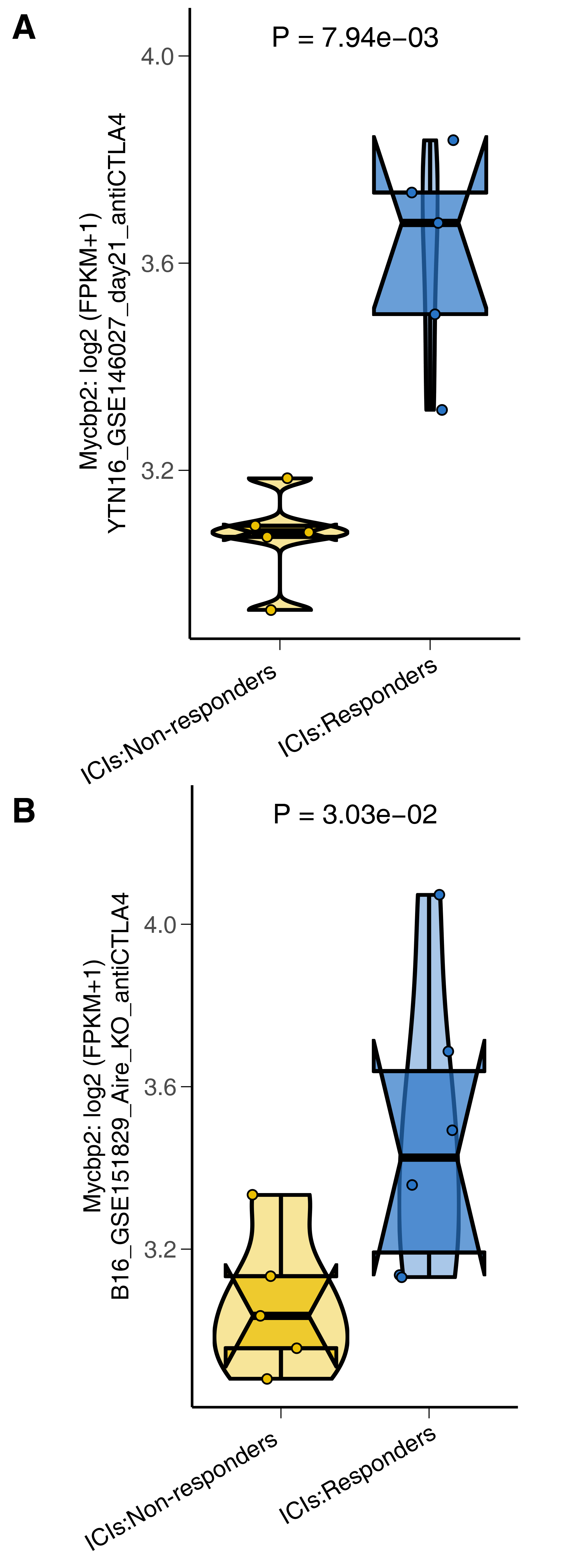

Supplement: Supplementary Figure 1 — The differences of the expression of the Mycbp2 between the ICIs-responders and ICIs-non-responders in the GSE146027 (A) and GSE151829 (B). [file Image_1.tif]

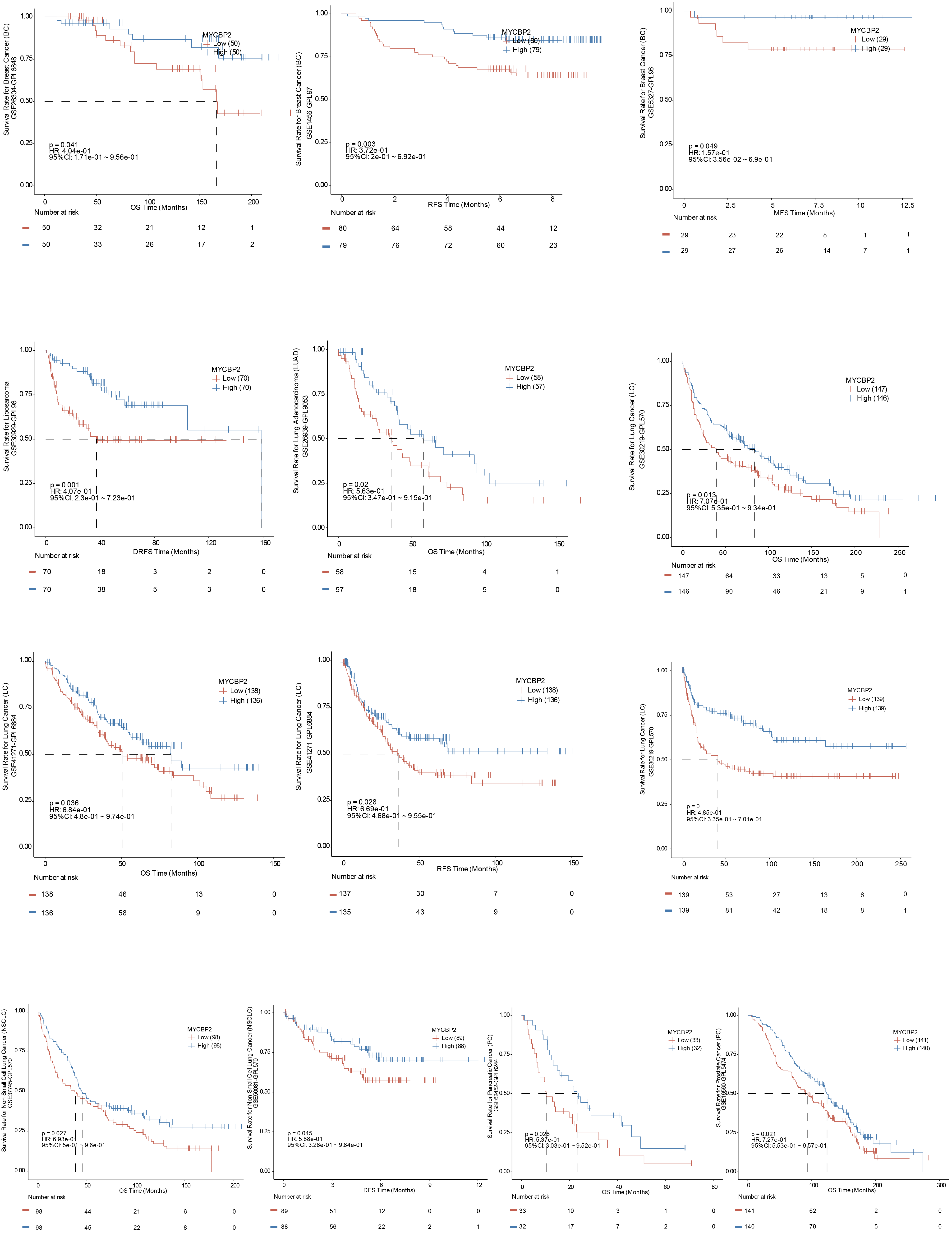

Supplement: Supplementary Figure 2 — The Kaplan-Meier curves depicting differences in the prognoses of cancer patients between the MYCBP2-High and MYCBP2-Low groups (GSE26304, GSE1456, GSE5327, GSE30929, GSE26939, GSE30219, GSE41271, GSE30219, GSE37745, GSE50081, GSE62452, and GSE16560). [file Image_2.tif]

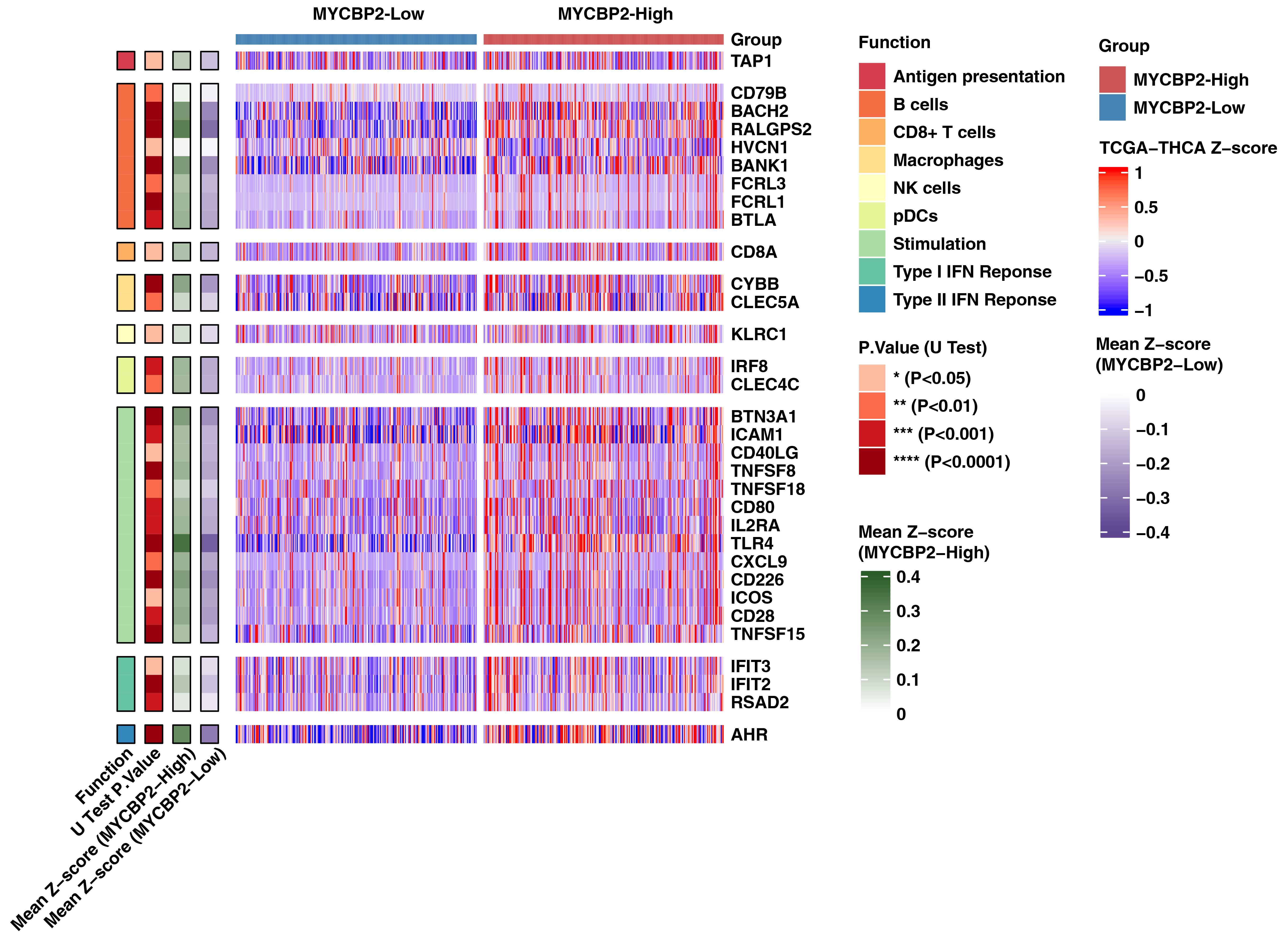

Supplement: Supplementary Figure 3 — Heatmap depicting differences in the expression of immune-related genes (antigen presentation, B cells, CD8+ T cells, macrophages, NK cells, pDCs, stimulation, and IFN responses) between MYCBP2-High and MYCBP2-Low patients in TCGA-THCA. [file Image_3.tif]
